# The Impact of Aging on Ocular Diseases: Unveiling Complex Interactions

**DOI:** 10.14336/AD.2024.0850

**Published:** 2025-09-23

**Authors:** Luling You, Yumeng Lin, Yizhuo Zheng, Zhongyu Han, Liuzhi Zeng, Haoran Chen

**Affiliations:** ^1^School of Medical and Life Sciences, Chengdu University of Traditional Chinese Medicine, Chengdu, China.; ^2^Health Management Center, Nanjing Tongren Hospital, School of Medicine, Southeast University, Nanjing, China.; ^3^Chengdu Women's and Children's Central Hospital, School of Medicine, University of Electronic Science and Technology of China, Chengdu, China.; ^4^Science Education Department, Chengdu Xinhua Hospital Affiliated to North Sichuan Medical College, Chengdu, China.; ^5^Department of Ophthalmology, Chengdu First People's Hospital, Chengdu, China.

**Keywords:** ageing, eye, age-related macular degeneration, age-related cataracts, glaucoma, diabetic retinal degeneration, dry eye disease

## Abstract

Aging is characterized by a progressive loss of physiological integrity, leading to impaired function and increased vulnerability to death. Aging is an important risk factor for eye diseases. The gradual deterioration of ocular tissue structure and function with age leads to the onset and progression of ocular diseases. During aging, ocular tissues such as the lens, vitreous and retina are affected by age-related changes, such as oxidative stress and protein accumulation in the lens leading to cataract formation, and a decline in retinal pigment epithelial cell function associated with macular degeneration. This article reviews the relationships between aging and ocular diseases, takes age-related macular degeneration, age-related cataracts, glaucoma, diabetic retinal degeneration, and dry eye disease as focal points, analyses the complex interactions between aging and ocular diseases, and describes the therapeutic options and potential targets for age-related ocular diseases.

## 1. Introduction

Aging is a natural process that involves a variety of cellular, molecular, and organ-level changes within the human body [1]. As life expectancy continues to rise across the global population, age-related diseases have emerged as a major public health concern. Among the most prevalent age-related changes are declines in organ function, including that of the eyes. The eyes play a critical role in daily life, and vision impairment or blindness can significantly impact a patient's ability to read, write, walk, and engage in other activities [2].

Ocular diseases, such as age-related macular degeneration (AMD), cataracts, and glaucoma, represent leading causes of vision impairment and blindness, imposing significant burdens on affected individuals and society. Given the increasing proportion of elderly individuals in the global population, the prevalence of eye diseases is anticipated to rise in the coming decades.

**Figure 1.**
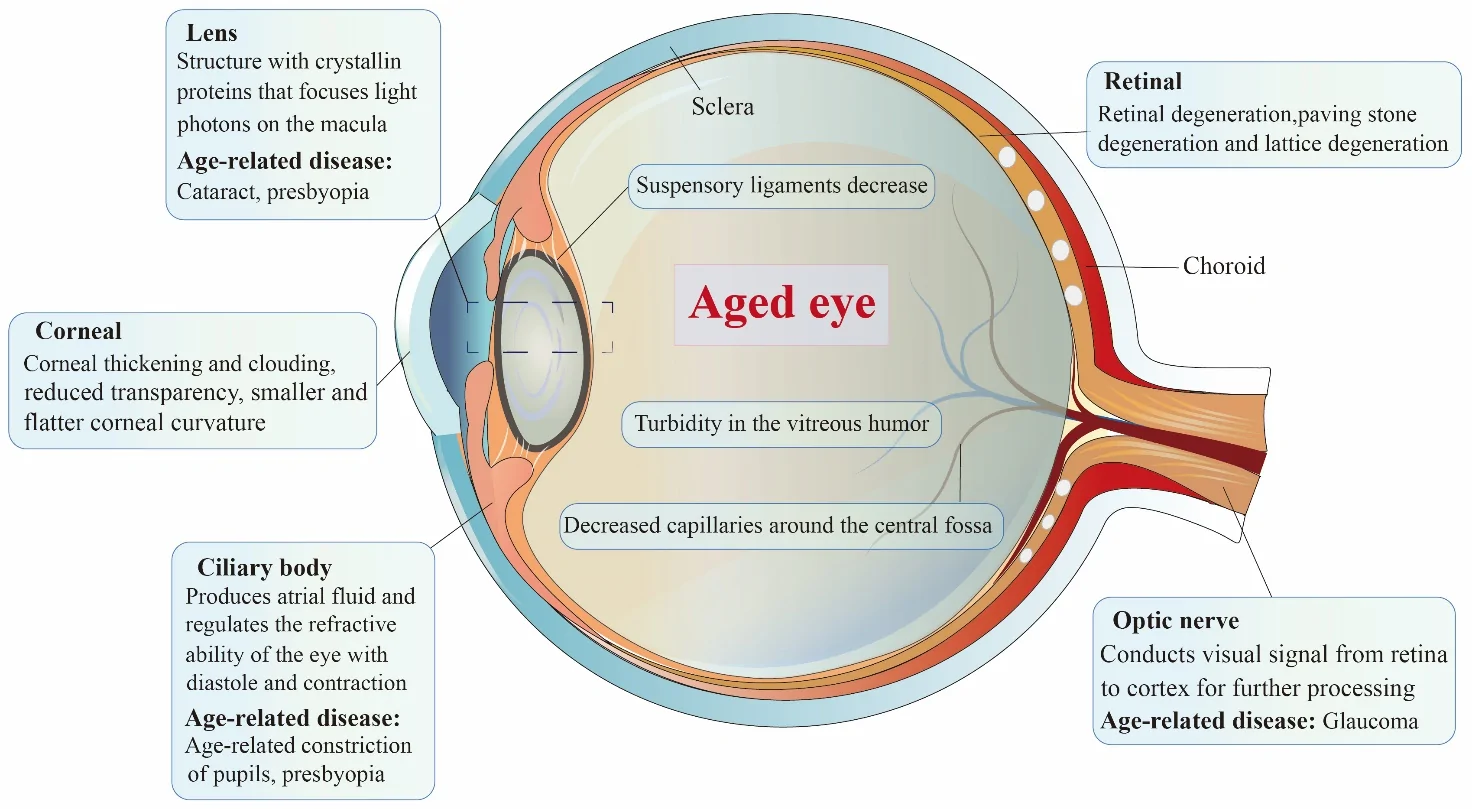
**The effects of ageing on the eyes**. As age increases, the lens thickens and hardens, leading to protein aggregation and clouding, which causes cataracts. The ciliary zonules weaken, the retina degenerates along with optic nerve cells, and retinal blood vessels may sclerose. Additionally, the vitreous becomes cloudy, and intraocular pressure rises. Ageing deteriorates both the structure and function of the eye, heightening susceptibility to various eye diseases.

The aging process has comprehensive effects on various aspects of eye structure and function, including the lens, retina, and optic nerve (Fig. 1). Perturbations in visual acuity, contrast sensitivity, and color discrimination arise as age-related changes unfold, ultimately culminating in diminished visual perception and compromised quality of life [2]. Elucidating the intrinsic mechanisms governing the interplay between aging and ocular diseases is highly important for the development of efficacious preventive and therapeutic modalities.

In this review, we explore AMD, cataracts, glaucoma, diabetic retinal degeneration, and dry eye disease (DED) as our focal points to investigate the intricate interplay underpinning aging and ocular diseases. Our analysis concentrates specifically on elucidating the molecular and cellular events that underlie the initiation and progression of these ocular diseases. Moreover, we aim to discuss prevailing therapeutic strategies while concurrently highlighting emerging interventions aimed at ameliorating the vision impairment associated with age-related eye diseases.

## 2. Methods

We conducted a systematic search of articles published up to June 25, 2024, of online databases including PubMed, EMBASE, and Web of Science, without back date restriction or language limitations. The search terms used were "(aging OR cellular senescence) AND (age-related macular degeneration OR AMD) ", "(aging OR cellular senescence) AND (cataract OR lens opacity)", "(aging OR cellular senescence) AND (diabetic retinopathy OR DR)", "(aging OR cellular senescence) AND (dry eye disease OR DED OR dry eye syndrome OR DES)", "(aging OR cellular senescence) AND (glaucoma)".

We screened 1,200 published papers based on title and abstract relevance. Eligible articles met the following inclusion criteria: (1) Focused on the impact of aging on eye diseases; (2) Employed study designs such as cohort studies, cross-sectional observational studies, or population studies with validated data; (3) The results showed that the occurrence of eye diseases was related to age. Exclusion criteria were: (1) studies involving organisms other than humans or mice; (2) conference abstracts; (3) studies not directly related to aging. Ultimately, 150 papers were included in our review.

The selected studies were critically evaluated for methodological quality and relevance to the topic. We assessed the strength of evidence provided by each study, ensuring that the review reflects a balanced and comprehensive understanding of how aging impacts various ocular diseases.

## 3. The effects of aging on the eye

Aging represents an inevitable physiological process characterized by a gradual deterioration of an organism's physiological functions, accompanied by discernible changes in both appearance and functionality [3]. The major molecular and cellular hallmarks of normal aging include genomic instability, telomere attrition, epigenetic alterations, loss of proteostasis, disabled macroautophagy, deregulated nutrient sensing, mitochondrial dysfunction, cellular senescence, stem cell exhaustion, altered intercellular communication, chronic inflammation and dysbiosis [4].

**Figure 2.**
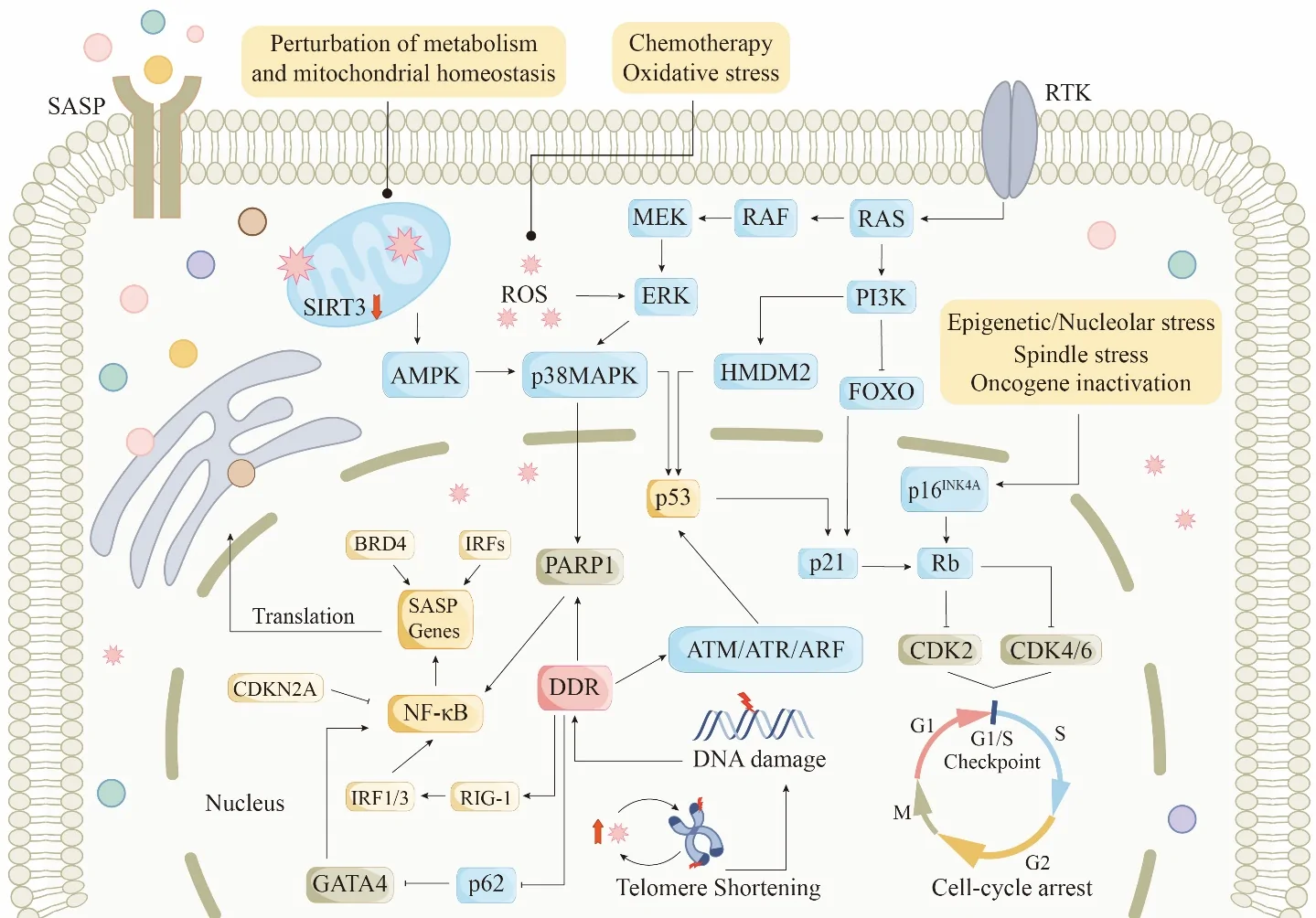
**Mechanisms associated with aging**. Senescence is associated with many mechanisms, including telomere damage and shortening, DNA damage response, cellular senescence, oxidative stress, mitochondrial dysfunction, and activation of signaling pathways. Collectively, these processes contribute to senescence by inducing cell cycle arrest. Accumulation of DNA damage triggers activation of the p53 gene, which in turn induces the expression of the downstream effector p21. This process inhibits cell cycle progression and contributes to cellular senescence. Concurrently, the rise of ROS and the reduction of mitochondrial SIRT3 activate the p38MAPK pathway, which subsequently enhances p53 activity, causing cellular senescence. Additionally, DDR activates GATA4, which subsequently upregulates NF-κB. This activation of NF-κB directly drives cellular senescence. The elevated NF-κB levels increase the transcription of SASP genes during DDR. Both cellular senescence and tissue aging are accelerated by the following increase in SASP factors' translation and release. In senescent cells, the expression of the p16 gene gradually increases, along with changes in the activity of Rb proteins, and this change affects cell cycle progression and leads to cellular senescence. Abbreviation: DDR (DNA damage response); ROS (reactive oxygen species); SASP (senescence-associated secretory phenotype).

The fundamental underlying factor of aging is the progressive accumulation of genetic material damage, specifically lesions and mutations within DNA, as well as the presence of misfolded proteins that impair cellular function and organelles [3, 5]. This accumulation of dysfunctional cells, resulting from alterations in cell fate, disrupts homeostatic processes and ultimately leads to a decline in organ function and the onset of various diseases. These cumulative processes, operating at the molecular level, contribute to the molecular damage that drives the aging process (Fig. 2).

The human eye is a remarkable organ, with light entering through the cornea and lens to focus on the retina. Photoreceptor cells then perform the crucial task of converting light signals into nerve impulses, which are subsequently transmitted via the optic nerve to the visual cortex of the brain for image formation and analysis [6]. Regrettably, the aging process can lead to the development of various ocular diseases, such as DED and cataracts, resulting in impaired vision [7]. In the following section, we provide a concise overview of the effects of aging on the eyes.

### 3.1. Lens aging

The lens, one of two transparent tissues in the body, plays a vital role in focusing light onto the retina. As individuals age, the thickness and weight of the lens increases [8]. The α-, β-, and γ-lens proteins, the key proteins in the lens, undergo age-related changes such as oxidation, deamination, truncation, glycation, and methylation [9, 10].

The outermost layer of the lens is a thin film capsule dominated by glycoproteins and collagen. New laminin fibres continue to form under the lens capsule epithelium, with these fibres primarily deposited in the horizontal direction of the lens. The aging lens gradually accumulates more layers of fibres, but the growth rate decreases [11]. As a result, the anterior and posterior diameters of the lens increase by approximately 50%, leading to increased light scattering rather than focalization on the retina. This condition results in a loss of the eye's ability to adapt and difficulty in seeing nearby objects, leading to the development of presbyopia [12].

Furthermore, the lens nucleus undergoes compression and hardening, with the lens proteins taking on a yellow to brown hue [13]. This process causes the lens to gradually lose its transparency, becoming opaque over time. The resulting cataracts are the leading cause of blindness worldwide [14, 15].

### 3.2. Retinal aging

The retina is a transparent membrane in the inner layer of the eye wall, consisting of the pigment epithelium and the sensory layer of the retina. As individuals age, various degenerative changes, including typical retinal peripheral capsular degeneration (TPCD) and paving stone (cobblestone) degeneration, occur in the peripheral retina [16]. The diffuse thickening of the inner retinal limiting membrane, coupled with a decrease in peripheral retinal nerve cells and glioblast proliferation, may be associated with choroidal vascular insufficiency.

After the age of 30, there is a progressive loss of optic rod cells in the macula. This results in shortening of the outer segments of the optic rods, which detach from the microvilli of the pigment epithelium. As a result, there is a reduction in retinal purplish matter and a decrease in the eye's sensitivity to low light compared with good light conditions [16].

Compared with adolescence, aging also affects the retinal vasculature, leading to a widespread loss of peripheral capillaries and a decrease in the number of capillaries surrounding the central fossa in older adults. Ocular blood flow and its regulation also change with aging, with older retinas showing an average 20% reduction in macular blood flow. When lesions occur in the macula, AMD becomes more likely [16, 17].

### 3.3. Corneal aging

The cornea is the main refractive component of the human visual system and provides focusing ability [18]. It is often considered a viscoelastic material, exhibiting both sticky and elastic properties. Structurally, the normal cornea consists of five layers, progressing from the outer surface to the inner surface: the multilayered epithelium, Bowman's layer, interspersed mesenchyme with keratocytes, Descemet's membrane, and the unilayered endothelium.

Aging manifests certain changes in the cornea. Calcified deposits tend to accumulate at the periphery of Bowman's layer, and the thickness of Descemet's membrane increases [18, 19]. These age-related alterations result in corneal thickening and cloudiness, leading to reduced transparency. Additionally, the cornea may undergo changes in size, becoming smaller and flatter in curvature and exhibiting uneven horizontal to vertical variations. Consequently, hyperopia (farsightedness) and astigmatism may occur. The emergence of a grey ring-shaped turbid area, approximately 1-2 mm wide, near the edge of the bulbar conjunctiva, referred to as arcus senilis, is common in elderly individuals, with an incidence rate as high as 40% [20].

Corneal endothelial cells, which are responsible for maintaining the structural integrity of the cornea, are terminally differentiated cells. With age, the density of these cells decreases, increasing the susceptibility of elderly individuals to various factors that can cause endothelial injury [21].

### 3.4. Ocular aging and the immune microenvironment

Aging significantly impacts the immune system's regulatory processes, leading to increased vulnerability to infections. The innate immune response is weakened, with a noticeable decline in functions such as the migration of neutrophils, the ability to kill microbes, and the phagocytic capacity. Additionally, the activity of macrophages, which are crucial for immune defence, is compromised, particularly their capacity to produce cytokines [22, 23].

Furthermore, the effectiveness of natural killer cells, which play a pivotal role in combating viral infections and tumor cells, is diminished. This reduction is exemplified by a decrease in the levels of various cytokines, such as TNF-alpha, IL-2, IL-12, and IL-2R, which are critical for coordinating immune responses [24].

In the adaptive immune system, aging leads to decreased B-lymphocyte production, diminished CD4+ T-cell support, and compromised antibody responses. Additionally, there is a notable increase in regulatory T cells (Tregs), signaling an overall shift toward immune suppression with age [25].

Due to the blood-ocular barrier, absence of direct lymphatic drainage, and a microenvironment enriched with immunosuppressive molecules, humans obtain ocular immune privilege, a state characterized by a restricted immune response. These features collectively safeguard vision by preserving tissue homeostasis and functionality [26].

Neutrophils, which are pivotal in mediating acute inflammation and serve as the primary defense against bacterial infections, exhibit age-related functional changes that impact ocular health [27]. In the conjunctiva, aging is associated with elevated levels of IL-8, a proinflammatory cytokine produced by neutrophils and epithelial cells. This increase may impair ocular surface functionality, increasing the susceptibility of the eye to infections [28]. Furthermore, the disruption of neutrophil homeostasis on the ocular surface contributes to age-related proinflammatory alterations, underscoring the relationships among aging, neutrophil function, and ocular inflammation.

The complement system is a unique component of the innate immune system and underpins the humoral immune system. Notably, it has been implicated in the pathogenesis of dry eye in mice, where it enhances autoantibody-induced inflammation of the ocular surface [29]. Aging is associated with elevated levels of complement Factors 3 and 4 across various tissues and sera, suggesting a systemic modulation of complement activity with age [30-32]. Given the established link between complement dysregulation and AMD, it is plausible that such dysregulation also contributes to the decline in ocular mucosal immune function observed in elderly individuals [33].

Antigen-presenting cells (APCs) represent a specialized class of cells that process antigens and present them to T cells as antigenic peptide-MHC molecular complexes. Recent studies have highlighted the heightened susceptibility of mucosal APCs to the aging microenvironment compared with their cutaneous counterparts [34]. The aged ocular environment is enriched with CXCL13, IL-1β, MHC II, IL-12, and IFN-γ, all of which can influence the activity of APCs present in the conjunctiva, limbus, and cornea [35]. Compared with their younger counterparts, these ocular APCs exhibit age-related changes, with senescent APCs expressing higher levels of CD86 and displaying increased phagocytic activity compared to their younger counterparts.

In aging tissues and organs, there is an increase in both the number and activity of CD4+ and CD8+ T cells, leading to increased cytokine release and participation in pathological reactions [35-37]. Notably, aging increases T-cell reactivity even towards non-antigens [38]. The predominant cellular composition of the human conjunctiva comprises lymphocytes, followed by monocytes and neutrophils, with CD8a/b cells being the prevailing subtype and CD4+ T cells constituting the next most abundant population [39].

Furthermore, the increased infiltration of CD4+ cells in the conjunctiva of senescent mice, along with their secretion of IFN-γ, indicates enhanced Th1 reactivity within the conjunctiva, lacrimal glands, and draining lymph nodes, which may contribute to the development of dry eye [35]. Dysfunction of Tregs due to aging has been suggested, as evidenced by disruptions in the balance between effector and regulatory CD4+ T cells and alterations in the Th1/Treg or Th17/Treg ratios with age, although the precise underlying mechanisms remain elusive [40, 41].

In a similar manner, aging has a profound effect on B lymphocytes. While the number of memory B-cells tends to increase with age, there is a concurrent decline in B cell production and compromised immune responses, potentially contributing to the development of autoimmune diseases [42]. The expression of the chemokine receptors CXCR3 and CD11c, which are associated with homing to inflamed sites, is more pronounced in the LM B cells of older individuals than in those of their younger counterparts. These findings suggest the potential migration of these cells to inflamed tissues, where they may secrete proinflammatory mediators, leading to localized inflammation [43].

Within the lacrimal gland, aging is accompanied by an increase in CD4+ and CD8+ T and B cells, as well as a decrease in activated dendritic cells. These observations suggest that age-related autoimmunity contributes to the development of DED over time [44]. Furthermore, in the senescent lacrimal gland, there is an increase in B1B cells (CD19+B220-) and a decrease in CD19-B220+ cells, whereas marginal zone-like B cells accumulate. This finding implies the involvement of humoral immunity in ocular surface inflammation associated with aging [45].

## 4. Pathological role of aging in ocular diseases

**Figure 3.**
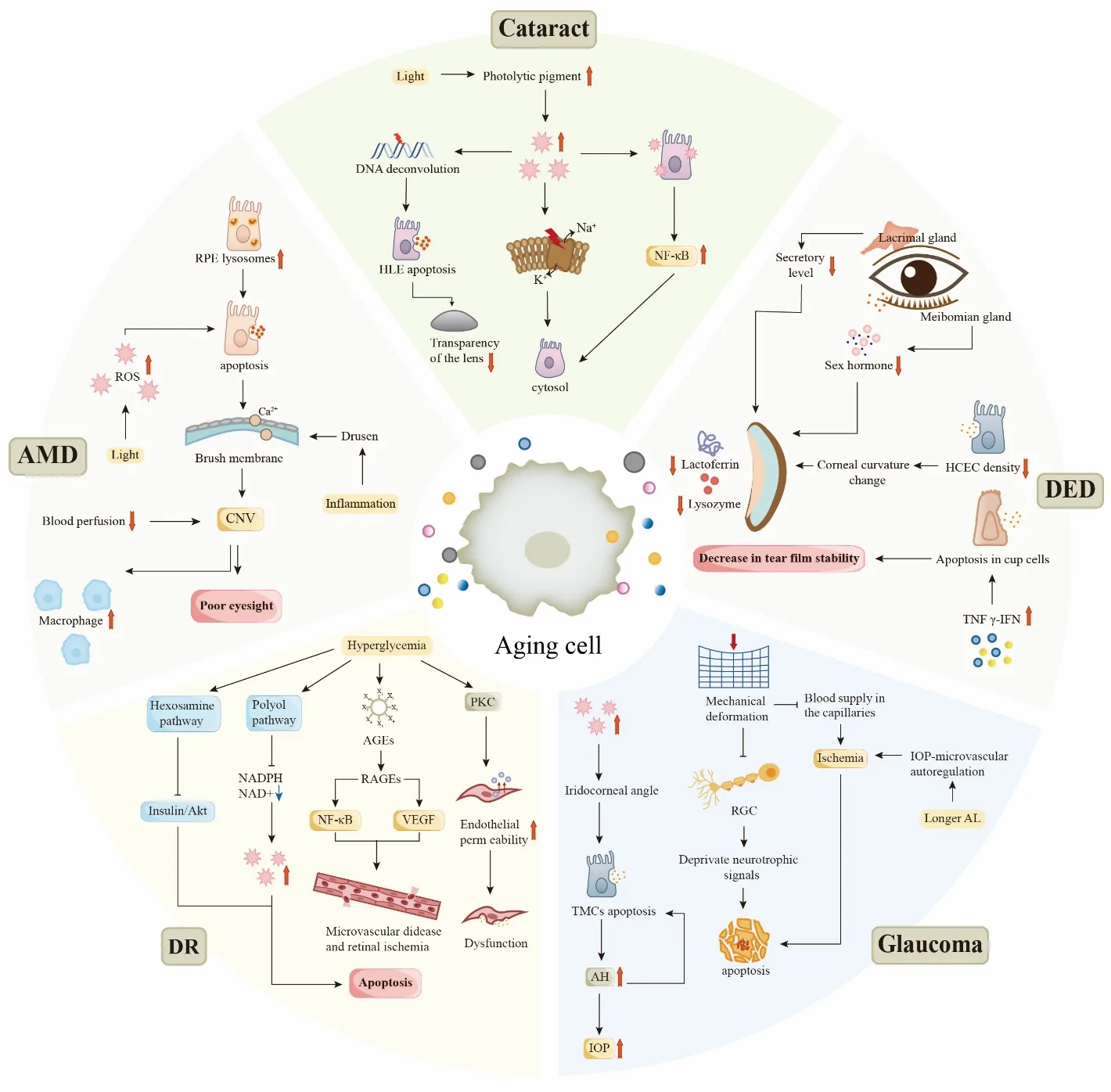
**The relationship between ageing and eye diseases**. As ageing progresses, there is a notable decline in ocular function. AMD, glaucoma, dry eye syndrome, and DR are commonly associated with this process. These diseases arise from various mechanisms, including cellular ageing, oxidative stress, and the release of inflammatory mediators. Ageing and photostimulation lead to excessive ROS production in the lens epithelium, resulting in ROS accumulation that causes DNA double-strand breaks and oxidative damage. This damage deactivates lens epithelial cells, leading to apoptosis and cataract formation. Additionally, ROS-induced apoptosis of RPE cells fosters CNV, contributing to AMD. ROS also promote apoptosis of TMCs, increasing AH accumulation and IOP, which may result in glaucoma. The density of HCECs gradually decreases and the level of lacrimal secretion decreases with age, resulting in reduced tear film stability and ultimately leading to dry eye. Abbreviation: AGEs (advanced glycation end products); AH (aqueous humor); AL (Axial length); CNV (choroidal neovascularization); HCEC (human corneal endothelial cells); HLE (human lens epithelial cells); IOP (intraocular pressure); NAD (nicotinamide adenine dinucleotide); NADPH (nicotinamide adenine dinucleotide phosphate); PKC (protein kinase C); RAGEs (Receptors for advanced glycation end-products); RGCs (retinal ganglion cells); ROS (reactive oxygen species); RPE (retinal pigment epithelium); TMCs (trabecular meshwork cells); VEGF (vascular endothelial growth factor).

As age increases, the structure and function of the eye significantly changes, increasing the risk of a variety of eye diseases (Fig. 3). The aging process involves the progressive loss of lens clarity, which is precipitated by the aggregation of lens proteins that culminate in cataract formation. Additionally, aging is associated with elevated intraocular pressure, thereby increasing the risk of glaucoma. Aging leads to degeneration of the macular area in the retina, which is the primary cause of AMD.

Moreover, in individuals with diabetes, aging exacerbates microvascular damage within the retina, exacerbating the onset and progression of diabetic retinopathy (DR), potentially resulting in severe visual impairment. Concurrently, declining tear gland function manifests as ocular surface dryness and discomfort, precipitating dry eye syndrome and increasing the susceptibility of the eye to inflammation and damage.

### 4.1. Age-related cataracts (ARCs)

ARCs, commonly referred to as senile cataracts, are the most prevalent form of cataracts and are typically observed in individuals over the age of 50, particularly among middle-aged and elderly individuals. The incidence of this condition significantly increases with advancing age, displaying a clear correlation with lens aging [46]. The lens is a transparent structure located behind the iris and in front of the vitreous body and retina. Its shape, clarity, and refractive index enable it to focus light onto the retina. With increasing age, changes in the chemical composition, content, and metabolism of the lens occur, contributing to its aging process. These alterations lead to diminished transparency and optical clarity, culminating in the formation of cataracts [47, 48].

ARCs commonly cause blurred vision, sensitivity to glare, halos around lights, reduced visual acuity in dim or nighttime environments, reduced contrast sensitivity, and double vision perception in one eye. ARCs can be categorized into three distinct types based on the location of clouding within the lens: nuclear, cortical, and posterior subcapsular cataracts [49].

Free radicals may play a role in lens aging. With aging, the stimulation of the eyes by sunlight intensifies, with the exposure to ultraviolet radiation increasing annually. This can lead to cellular dysfunction through metabolic processes and external stimuli that cause cells to continuously produce free radicals.

With aging, the body's ability to produce antioxidants decreases, creating an imbalance between free radicals and the antioxidant system. The accumulation of free radicals within the lens escalates as a result, triggering oxidative stress. This oxidative stress can affect the cell membrane and inflict harm upon DNA and various intracellular proteins. In the end, it results in the lens losing its clarity and becoming cloudy [50]. ARCs are mostly caused by oxidative damage [51].

There are notable alterations in the amounts of lens structural proteins (crystallins) that correspond with the aging of the lens. Approximately 90% of the soluble proteins in the vertebrate lens are composed of a class of proteins called crystallins. Mammals possess three different immunological crystallins, known as α, β, and γ crystallins [9]. Lens crystallins are nonrenewable, long-lasting proteins. As a result, they are very vulnerable to the age-related buildup of advanced glycation end products (AGEs) and glycosylation [52].

Significant changes in the human lens occur with aging, such as reduced glutathione levels, elevated production of ROS, compromised antioxidant defences, and elevated levels of redox-active metal ions that trigger protein glycation and the breakdown of glucose and ascorbic acid UVA-mediated free radicals, which also draw metal ions to attach to them and cause further protein oxidation and modification, can be produced by glycosylated lens proteins. Both the development of diabetes and aging exacerbate this vicious cycle [53]. Lens clouding results from changes in the transmittance and refractive power of the lens caused by changes in the concentration of structural proteins in the lens.

In addition to affecting lens proteins, aging likewise affects lens epithelial cells. The processes of equatorial epithelial cell division and the development of generations of differentiated fibroblasts are the mechanisms by which the human lens develops. Additionally, the fibrous mass of the lens is periodically supplemented with new fibres. Lens homeostasis and transparency are preserved by epithelial cells and their active transport systems [54]. Oxidative damage and aging affect the lifetime development process of lens epithelial cells [55].

The aged cataract lens epithelium presented significantly lower activities of succinate dehydrogenase, lactate dehydrogenase, glucose-6-phosphate dehydrogenase, and adenosine triphosphatase. This result suggests that the antioxidant capacity of the lens decreases significantly with age, leading to a lack of energy supply, which in turn causes structural damage to the lens epithelium and ultimately causes clouding of the lens [56].

Glutathione peroxidase 1 (GPX1) is expressed in lens epithelial cells and cortical fibrocytes. Compared to catalase, GPX1 exhibits a superior capacity to decompose H_2_O_2_ and alleviate oxidative stress, playing a critical role in maintaining the transparency, optical quality, and balance of aging lens [57].

Recent studies reveal that aging-associated proteins in the anterior lens capsules (ALCs) of cataract patients, including p53, total laminins (LM), LM-α4, and transforming growth factor-β1 (TGF-β1), exhibit a positive correlation between ARCs levels. The process of aging is associated with a reduction in matrix metalloproteinase-9 (MMP-9), which in turn increases levels of LM-α4 and TGF-β1. This upregulation activates the p38 MAPK signaling pathway, thereby promoting the development of ARCs. These findings suggest that LM-α4 and its regulatory pathways represent promising targets for drug development aimed at preventing and treating ARCs [58].

Glutathione S-transferase Mu 3 (GSTM3) plays a crucial role in protecting the lens from oxidative stress. In ARC lens tissues, expression of GSTM3 is notably reduced. This decrease is associated with low levels of acetylated H3 at the GSTM3 promoter and elevated levels of trimethylated H3K9, suggesting that promoter hypermethylation and altered histone modifications contribute to diminished GSTM3 expression, thereby facilitating progression of ARCs [59].

### 4.2. Glaucoma

The age-standardized prevalence of glaucoma, a chronic degenerative visual neuropathy, is the second most common cause of blindness globally. Currently, approximately 11.2 million people are experiencing binocular blindness due to glaucoma worldwide. It is projected that by the year 2040, an additional 118.8 million individuals will develop this condition [60, 61]. The prevalence of glaucoma is now 2. 86% worldwide.

Based on the condition of the iridocorneal angle, glaucoma can be divided into two categories: primary open-angle glaucoma and primary closed-angle glaucoma. Glaucoma is typically linked to ocular hypertension. Whereas the latter involves the forward bending of the peripheral iris, causing a progressive relative closure that obstructs aqueous flow and increases intraocular pressure (IOP), the former is associated with an unobstructed iridocorneal angle and no other discernible circumstances that may obstruct aqueous flow [62]. The ultimate consequence is irreversible blindness, which frequently manifests as bilateral lesions and is caused by the degeneration of RGCs and their axons as well as weakening of the neuroretinal margin of the optic nerve head [63].

One of the most important risk factors for glaucoma is aging, or the stable departure of cells from the cell cycle as a result of stress and injury. Cellular senescence is a characteristic of aging. In glaucoma, cellular senescence, oxidative stress, DNA damage, mitochondrial dysfunction, problems in autophagy and microphagy, and epigenetic changes are some of the possible pathways of aging.

#### Trabecular meshwork aging

Age and pressure-related accumulation of senescent trabecular meshwork cells (TMCs), RGCs, and vascular endothelial cells has been shown to potentially exacerbate glaucomatous diseases. A significant part of the aqueous outflow channel, the trabecular meshwork, is essential for preserving a normal IOP [64]. Oxidative stress, which is characterized by an imbalance between oxidative and antioxidant effects, is a direct risk factor for high IOP in glaucoma patients.

Increased resistance to atrial aqueous outflow and increased pathological IOP are the final results of trabecular meshwork cell malfunction, which is caused by oxidative stress in the cells. This stress can also result in extracellular matrix (ECM) deposition and degradation, autophagy, and senescence [65].

Oxidative stress contributes to the premature aging of TMCs. First, aging diminishes the expression of nuclear-encoded genes involved in oxidative phosphorylation, leading to mitochondrial dysfunction and an accumulation of ROS. Second, heightened ROS levels can cause oxidative damage to mitochondrial DNA (mtDNA) and proteins, exacerbating cellular harm and inciting further cellular senescence [66]. A reduction in Prdx6 will result in an increase in ROS levels, which will in turn lead to the deleterious effects of overstimulation of p21 and p16 [67]. Furthermore, the downregulation of miR-106a may facilitate the development of certain characteristics associated with senescent cells by upregulating p21 in oxidative stress-induced SIPS in TMCs [68]. This heightened oxidative stress in TMCs leads to the malfunction of trabecular meshwork cells and the emergence of senescence markers.

The overexpression of ATF4 has been demonstrated to induce the expression of C/EBP homologous protein (CHOP) and promote TMCs apoptosis. This promotes the production of inflammatory cytokines, which may lead IOP increasing [69].

#### Retinal ganglion cell apoptosis

Ganglion cells and their axons are the keys to glaucomatous optic nerve damage [70]. RGCs form the optic nerve directly to transfer visual signals. Ganglion cells are lost throughout life in normally aged adults; approximately 4,900 axons are lost annually. Research has indicated that in healthy individuals, the thickness of the parafoveal retinal nerve fibre layer decreases by approximately 0. 20 μm annually and the thickness of the ganglion cell layer decreases by approximately 1. 22 μm annually. The amplitude of pattern electroretinograms decreased with age in a study including rats that ranged in age from 18 to 59 weeks, indicating that ganglion cell function decreases with normal aging [71, 72].

Overexpression of OPA1 reduces RGCs cytotoxicity and apoptosis while enhancing mitochondrial fusion and autophagy, thereby promoting RGCs survival [73]. The anti-aging molecule Sirt6 is highly expressed in RGCs. Global or targeted knockdown of Sirt6 results in the degeneration of RGCs and their axons, thereby contributing to RGC senescence. Therapeutic targeting of Sirt6 through AAV2-mediated gene delivery effectively mitigates RGC degeneration induced by elevated IOP [74]. Therefore, promoting the expression of OPA1 and Sirt6 may help to delay the development of glaucoma.

Moreover, in a mouse model of elevated IOP and in human eyes with primary open-angle glaucoma (POAG), the SIX6 risk variant (rs33912345, His141Asn) has been implicated in POAG pathogenesis through the upregulation of p16^INK4a^ expression, which accelerates retinal ganglion cell senescence in the adult retina [75]. Another study demonstrated that acute IOP induced ischemia enhances TBK1 and p16 expression through the Akt-Bmi1 phosphorylation pathway, thereby accelerating RGC aging [76].

These findings suggest a close association between RGC aging and death with the pathogenesis of glaucoma. Furthermore, modulating the expression of age-related proteins appears to be crucial for mitigating glaucoma progression.

#### Mitochondrial dysfunction

Optimal neuronal function crucially depends on mitochondria. Aging processes typically lead to a progressive decline in mitochondrial efficacy. In the context of glaucoma, patients often exhibit compromised mitochondrial function in RGCs, which is attributed to a diminished oxygen supply [66].

Cellular OS initiates a cascade in which dysfunctional mitochondria exacerbate endoplasmic reticulum (ER) stress, leading to increased ROS levels within cells [77]. Elevated ROS levels induce mitochondrial damage and disrupt normal autophagic processes, culminating in exacerbated mitochondrial dysfunction, cellular impairment, and ultimately, apoptosis, underlying the pathogenesis of glaucoma [78, 79].

Abnormal autophagic activity is typically linked to P53 overexpression, the inhibition of mitochondrial transport, and the activation of E3 ubiquitin ligases. These events ultimately prevent damaged mitochondria from being cleared from the cell, which results in cellular failure [80].

In a range of animal models of neurodegenerative disorders, mitochondrial uncoupling protein 2 (UCP2) has been shown to reduce oxidative stress and neurological damage [81]. Hass et al. established an elevated IOP model in UCP2 knockout animals by introducing microbeads into the anterior chambers. They reported that specific deletion of RGC-associated UCP2 reduced ROS-induced protein alterations and prevented RGC death. The absence of UCP2 promoted mitochondrial autophagy, safeguarding RGCs by eliminating dysfunctional mitochondria before they could compromise cellular integrity. The identification of UCP2 in glaucoma suggests that UCP2 is a promising therapeutic target for enhancing mitochondrial autophagy, potentially mitigating RGC loss under conditions of elevated IOP. RGC destruction is the fundamental cause of irreversible loss of visual function [82].

In a rat model of chronic hypertension glaucoma, the overexpression of Parkin was found to increase lysosome-associated membrane protein 2 levels concurrently with increased IOP, triggering mitochondrial autophagy and reducing RGC mortality [73].

According to Hu et al., RGC death is decreased by the overexpression of Parkin and optic nerve atrophy protein 1, indicating that RGCs are protected from damage by the clearance of damaged mitochondria [83]. In summary, Parkin promotes mitochondrial autophagy by targeting damaged mitochondrial degradation, thereby maintaining the health and function of RGCs, making it one of the potential targets for therapeutic intervention in glaucoma.

### 4.3. AMD

AMD is a multifaceted ocular condition characterized by photoreceptor death, and degeneration of the retinal pigment epithelium (RPE) and choriocapillaris (CC), primarily affecting the macular region of the retina. This pathology results in visual distortion, central vision loss, and diminished visual acuity.

AMD is the leading cause of blindness, with a 25% and 8% risk of early and late-stage disease, respectively, among individuals over 75 years of age. Projections suggest a global AMD incidence of 288 million individuals by 2040 [84]. While the precise aetiology remains elusive, known risk factors include smoking, advanced age, genetic predisposition, and hypertension [85].

#### SASP

Numerous inflammatory cytokines, chemokines, proteases, growth factors, and matrix modelling factors are secreted by senescent cells. This collective secretion, termed the senescence-associated secretory phenotype (SASP), profoundly alters local tissue micro-environments, contributing to chronic inflammation and carcinogenesis [86].

In the context of AMD, senescence-associated pathways such as the SASP may exert a pivotal influence. Senescent endothelial cells (ECs), for example, can release IL-1β, stimulating the p21/p53 pathway and augmenting the SASP [87]. Additionally, members of the TGF-β family, vascular endothelial growth factor (VEGF), and chemokines such as CCL2 and CCL20 facilitate paracrine senescence, impacting adjacent normal cells. Proteolytic enzymes including matrix metalloproteinases (MMPs) are also integral components of the SASP, and potentially contribute to AMD pathophysiology [88].

Aging impairs the MMP pathway, which decreases the pool of active MMPs available for matrix disintegration. This leads to the buildup of denatured collagen in different cellular structures [89].

Notably, Hussain et al. observed reported levels of active MMP-2 and MMP-9 in AMD patients, underscoring the dysregulation of the MMP during disease progression [90]. However, Chau et al. found that plasma MMP-9 levels were higher in patients with early and neovascular AMD than in healthy controls [91]. Subsequent studies by Zeng et al. showed no significant difference in serum MMP-2 and MMP-9 levels between patients with early or neovascular AMD and healthy controls [92].

The disparate experimental outcomes may be attributable to discrepancies in the methodologies for sample collection and analysis. Firstly, the studies employed different patient cohorts, potentially influenced by variations in age, disease severity, or comorbid conditions. Secondly, the techniques and standards for MMP assessment may differ significantly. For instance, different immunoassay methods may differ in terms of reference standards, antibody affinity, and experimental conditions, which may impact the comparison of study results [93].

Iron levels increase during aging, and iron overload can lead to mitochondrial dysfunction. MtDNA released into the cytoplasm activates the cGAS-STING pathway, promotes SASP, and induces RPE cell senescence [94]. This suggests that the accumulation of iron is also one of the factors causing AMD.

#### Oxidative stress

One of the causes of AMD is oxidative stress. The progression of AMD was attenuated in OXYS rats subjected to chronic treatment with the mitochondria-targeting antioxidant SkQ1, as evidenced by reduced amyloid β levels and suppressed retinal mTOR activity [95].

In vitro studies using human RPE cells cultured to simulate oxidative stress and aging have revealed age-specific alterations in cytokine secretion. Elevated expression of IL-1ra alongside increased secretion of VEGF, IL-12, and IL-10 in aged RPE cells has been associated with diminished cellular function and increased susceptibility to cell death, demonstrating a link between oxidative stress and aging and the pathophysiology of AMD [96].

The RPE is the main site of AMD. Patients with dry AMD have significant levels of bone morphogenetic protein-4 (BMP4) expression in their surrounding ECM and RPE. BMP4, in conjunction with sustained oxidative stress, induces RPE senescence via the p53-p21(Cip1/WAF1)-Rb pathway. Mechanistically, BMP4 enhances oxidative stress-induced senescence by activating p53 and p21(Cip1/WAF1)-Rb signaling, while concurrently diminishing the level of phosphorylated Rb through the Smad and p38 signaling cascades. Therefore, BMP4 stands out as a pivotal factor in the pathophysiology of dry AMD associated with oxidative stress-induced senescence [97].

#### DNA damage response

Numerous investigations have revealed that the development of AMD is significantly influenced by the DNA damage response. Ineffective DNA repair mechanisms can lead to prolonged damage to both the nucleus and mtDNA, potentially contributing to their longevity. The process of aging, a significant risk factor for AMD, may be associated with the progressive accumulation of mtDNA damage [98].

The RPE emerges as a primary site of mtDNA damage due to senescence, with damage severity increasing with age. Comparative studies between AMD patients and non-AMD individuals have revealed significantly elevated mtDNA heterogeneity in the RPE of AMD patients [99]. Moreover, mtDNA damage in mitochondria from AMD donors is approximately eightfold greater than that in nuclear DNA [100]. This accumulation of mtDNA lesions underscores its pivotal role in AMD pathogenesis

The incidence of the mt4917A>G mutation in the NADH dehydrogenase gene and the mtA11812A>G mutation in the NADH-ubiquinone oxidoreductase gene was found to be significantly greater in AMD-affected eyes than in normal eyes by Kaarniranta et al [101]. Additionally, a strong correlation was observed between these mutations and the level of ROS in RPE cells. The burden of mtDNA damage is exacerbated by age-related reductions in key mtDNA repair enzymes such as PARP1, mutY isoforms, and nucleic acid endonucleases, which are primarily responsible for mitigating oxidized DNA damage within the nucleus [102].

Dry AMD is characterized by CC degeneration, which is characterized by RPE hypoxia and atrophy. Recent investigations have revealed a correlation between reduced mitochondrial content and depletion of respiratory chain-associated subunits within RPEs, which is concurrent with pathological features such as depigmentation and increased subretinal deposits. These findings suggest that mtDNA damage and subsequent dysfunction precipitate metabolic disturbances, disrupt intracellular calcium homeostasis, and perturb mitochondria-to-nucleus signaling pathways. Together, these disruptions significantly compromise functionality [103].

#### Mitochondrial autophagy damage

Autophagy, a crucial cellular metabolic process, underpins cellular homeostasis, differentiation, developmental processes, and survival. Accumulating evidence indicates that autophagic activity diminishes across various organisms with advancing age [104-106].

Investigations involving Caenorhabditis elegans, rodent models, and human cell lines revealed a decline in lysosomal protein degradation over time, leading to impaired autophagic flux. This impairment exacerbates cellular damage and contributes to the onset of age-associated diseases [107]. Research in mice has demonstrated that the age-related decline in the expression of autophagy-related genes, including ATG5, ATG7, and BECN1, is associated with the emergence of an AMD-like phenotype in RPEs. This phenotype is characterized by alterations in RPE structure, including thickening, hypertrophy or atrophy, pigmentation irregularities and the accumulation of oxidized proteins [108].

Autophagy is decreased when lipofuscin accumulates in the RPE, which is a characteristic of AMD senescence. High mobility group Box 1, a senescence marker raised in iPSC-RPEs in response to the processing of lipofuscin components, is elevated in response to endoplasmic reticulum stress or mitochondrial injury [109, 110].

The downregulation of autophagy, coupled with mtDNA damage and elevated ROS production, triggers RPE apoptosis or inflammatory apoptosis. This process releases inflammatory and angiogenic factors, thereby fostering neovascularization [111]. These events collectively precipitate RPE atrophy and localized inflammatory reactions within the retina.

#### Metabolic aging

Metabolic processes associated with aging may indirectly impact longevity. Metabolic stress can precipitate a cascade of detrimental effects, including inflammation, oxidative stress, mitochondrial dysfunction, endoplasmic reticulum stress, and impaired autophagy [112]. Furthermore, these metabolic stress-related phenomena are intricately linked to retinal aging and the pathogenesis of AMD [113].

PGC-1α (peroxisome proliferator-activated receptor gamma coactivator 1-α), a pivotal transcriptional coactivator, regulates genes involved in energy metabolism and mitochondrial biogenesis.

Macular pigment is a yellow deposit under the retina that is made up of lipids and proteins and is a hallmark of AMD. PGC-1α inhibition combined with a high-fat diet induces a severe phenotype similar to AMD in mice. Specifically, PGC-1α inhibition alone results in RPE and retinal degeneration, accompanied by grape-like deposition. Furthermore, Inhibition of PGC-1α in RPE cells leads to an accumulation of lipid droplets and increased lipid peroxidation. These observations underscore the critical role of PGC-1α in the regulation of lipid metabolism [114].

PGC-1α enhances mitochondrial function by upregulating antioxidant enzymes and modulating DNA damage responses, while also exerting a protective effect against cellular senescence and oxidative stress-induced degeneration in the aging RPE. Additionally, PGC-1α plays a critical role in the pathogenesis of AMD through its regulation of VEGF [115].

### 4.4. Diabetic retinopathy

With global aging trends, the prevalence of diabetes is projected to rise sharply. Estimates indicate that the number of adults living with diabetes worldwide has increased from approximately 415 million in 2014 to an anticipated 642 million by 2050 [116, 117]. DR, a prominent microvascular complication of diabetes mellitus, is a leading cause of vision impairment and blindness among diabetic patients. The early stages of DR are characterized by the development of microaneurysms and intraretinal hemorrhages [117].

Numerous studies have shown that aging and hyperglycemia are the main risk factors for DR because diabetes and aging are closely related. Diabetes-related diseases, such as cardiovascular disease and visual impairment, are similar to aging, and the molecular mechanisms of both conditions, such as cellular senescence and abnormalities in protein homeostasis, are highly similar and parallel to those of aging [118, 119].

Chronic hyperglycemia can inflict direct damage to the retina through protein glycosylation. In this state, glucose molecules interact with proteins, forming aldimines that undergo structural rearrangement to yield stable ketamine compounds. Over time, these compounds aggregate and cross-link, evolving into complex AGEs.

As AGEs cross-link with ECM components, they accumulate, compromising cellular elasticity. This accumulation leads to the deterioration of retinal vascular endothelial cells and basement membranes, ultimately inducing microangiopathy and retinal tissue ischemia [120].

Receptors for advanced glycation end-products (RAGE) are prevalent on retinal peripapillary and endothelial cells, and their expression is amplified under diabetic conditions. This heightened RAGE expression triggers an oxidative cascade and accelerates the secretion of inflammatory factors, notably VEGF. Elevated VEGF secretion, in turn, exacerbates damage to the retinal microvasculature and the blood–retinal barrier [120-122]. Therefore, RAGE inhibition could serve as a potential therapeutic strategy for DR.

#### Metabolic abnormalities caused by hyperglycemia

The normal structure and function of retinal cells are impacted by hyperglycemia because hyperglycemia affects their metabolic pathways, particularly the glucose metabolic pathway. The balance between oxidative phosphorylation and glycolysis is necessary to maintain normoglycemia. Dysregulated glycolysis results in the production of many intermediate metabolites, which can be transferred to various damage pathways, such as the polyol pathway, the hexosamine pathway, and the diacylglycerol-dependent protein kinase C-activated pathway [123].

Insulin-independent tissues, including the nerves, retina, crystalline lens, and kidneys, metabolize glucose into sorbitol via aldose reductase, which is subsequently converted to fructose [124]. This polyol pathway becomes hyperactive in diabetes, leading to the accumulation of sorbitol and fructose, which increases the intracellular osmotic pressure, causing osmotic damage due to water influx [125]. The activation of this pathway also depletes NADPH and NAD+, which are crucial for glutathione regeneration, thus weakening antioxidant defenses and promoting ROS overproduction, resulting in oxidative stress [126]. Evidence from animal studies suggests that aldose reductase inhibitors, such as ranestat, can mitigate this damage by inhibiting the polyol pathway. Notably, ranestat has been shown to be neuroprotective when used early in DR by preventing the upregulation of aldose reductase activity [127].

In the hexosamine biosynthetic pathway, glucose undergoes phosphorylation to become glucose-6-phosphate, which then participates in a series of enzymatic conversions to ultimately yield hexosamine [128]. Elevated blood glucose levels trigger increased activity within the hexosamine pathway, resulting in increased concentrations of uridine diphosphate N-acetylglucosamine (UDP-GlcNAc), a crucial substrate for protein glycosylation. This upregulation promotes the expression of glucosamine-6-phosphate N-acetyl-transferase (GNPNAT1), the rate-limiting enzyme in the hexosamine pathway, thereby further amplifying its metabolic flux [129].

However, this overactivation has deleterious effects. It impedes the neuroprotective action of the insulin/Akt signaling pathway and intensifies retinal neuronal cell death [130]. Moreover, it triggers O-GlcNAcylation and activates the NF-κB p65 subunit, thereby exacerbating retinal ganglion cell death in diabetic mice [129, 131]. Furthermore, this pathway mediates the glycosylation of p53 in retinal pericytes, promoting pericyte apoptosis and early vascular dysfunction in DR [123].

Diacylglycerol production increases during hyperglycemia, and the combined action of calcium ions and phospholipids activates protein kinase C (PKC). Activated PKC increases neovascularization, stimulates the production of multiple cytokines, such as platelet-derived growth factor and VEGF, and increases inducible NO, which damages endothelial cells, inhibits nitric oxide synthase, and reduces the ability of nitric oxide to dilate blood vessels. Additionally, it promotes endothelial cell dysfunction and inhibits Na+/K+-ATPase activity, which results in retinal vascular dysfunction [132, 133].

#### Oxidative stress and the inflammatory response

Chronic hyperglycemia exacerbates oxidative stress and the inflammatory response in retinal tissues, resulting in intra- and extracellular environmental perturbations. This accelerates retinal cell aging and apoptosis.

In a study examining oxidative stress markers in rats, diabetic subjects presented elevated levels of markers in retinal tissues compared with normal subjects and reduced antioxidant levels [134]. Prolonged hyperglycemia triggers an increase in electron transport chain flux, reducing mitochondrial superoxide levels and adenosine triphosphate production in the retina. This is primarily due to excess glucose absorption leading to pyruvate production. Concurrently, this aberrant metabolism incites ROS overproduction in the retina, causing mitochondrial structural and functional disorders, and inducing endothelial and pericyte cell apoptosis [135, 136].

In contrast, aging retinas also display diminished electron transport chain functionality alongside increased ROS levels, suggesting that aging may amplify oxidative stress, thereby facilitating DR progression [137].

Evidence from numerous studies highlights that those senescent cells, including endothelial cells and fibroblasts, exhibit increased secretion of inflammatory factors [138, 139]. Notably, following chemotherapy, senescent epithelial cells exhibit significant overexpression and secretion of IL-1β [140]. The level of NF-κB, a transcription factor closely associated with aging, increases with age. Hyperglycemia-induced accumulation of AGEs activates RAGE, which in turn stimulates NF-κB, leading to elevated proinflammatory cytokine expression [38].

A recent study demonstrated that N-acetylserotonin mitigates retinal ischemia-reperfusion injury in rats through the HMGB1/RAGE/NF-κB signaling pathway. This research highlights that the RAGE promoter contains NF-κB binding sites, indicating a potential positive feedback loop between RAGE and NF-κB [141]. Furthermore, Sohn et al. identified functional NF-κB binding elements within the RAGE gene, further supporting the complex interplay between the RAGE and NF-κB signaling pathways [142].

Concurrently, RAGE functions as an adhesion receptor on endothelial cells, facilitating leukocyte aggregation, inciting inflammatory responses and enhancing endothelial cell permeability. Activated monocytes release a cascade of inflammatory mediators, recruiting and activating additional cells, thereby exacerbating vascular wall damage and accelerating the aging and apoptosis of retinal cells [143].

DR is characterized by chronic, low-grade inflammation, suggesting that the increased risk of DR with increasing age may stem from the age-associated increase in inflammatory factor secretion [144].

### 4.5. DED

DED is a chronic disease of the ocular surface characterized by the primary symptom of ocular dryness. The condition results from a range of factors, often age-related, that cause abnormalities in the quantity, quality, and kinetics of tears, leading to an unstable tear film or an imbalanced ocular microenvironment.

Common symptoms include itchiness, foreign body sensation, and burning of the eyes, as well as photophobia, blurred vision, and fluctuating visual acuity [145]. Recent studies have linked DED to alterations in the ocular surface, immune-based inflammatory responses, apoptosis, and changes in sex hormone levels.

A meta-analysis revealed a significant association between aging and DED development [146]. After 55 years of age, ocular surface integrity and tear film stability progressively decline due to lacrimal gland fibrosis, meibomian gland dysfunction (MGD), decreased corneal sensitivity and cell density, impaired immune defences, and hormonal changes leading to local inflammation, conjunctival chafing, and lid abnormalities [147].

The ocular surface encompasses the corneal epithelium, conjunctival epithelium, and tear film. A well-functioning tear film is essential for maintaining the normal structure and function of the ocular surface epithelium. The mucin components secreted by the ocular surface epithelium play crucial roles in tear film composition. Therefore, there is a mutual dependency and interaction between the ocular surface epithelium and tear film [148, 149].

The composition and stability of the tear film significantly change with age, as evidenced by a shortened tear film break-up time (TBUT), reduced tear volume, and increased tear osmolarity [28, 150]. Age-related alterations in tear composition have also been reported, with increases in several interleukins (IL-4, IL-6, IL-8, IL-3, IL-1β, IL- 17A, IL-12, and IL-10), IFN-γ, and TNF-α. These changes may elicit an immune-based inflammatory response [25, 146].

In addition, human tear fluid has lower levels of the growth factor IGF-1, which is strongly associated with DED clinical symptoms [151]. Studies employing healthy female C57BL/6 mice aged 2 and 22 months have demonstrated age-dependent alterations in tear secretion, corneal sensitivity, and morphology. Aged mice exhibit increased tear production, diminished corneal sensitivity, thickening of the corneal stroma, and epithelial thinning [152].

Aging is associated with MGD, characterized by impaired function of the large sebaceous glands on the eyelids, which are responsible for the secretion of cytolipids that prevent tear evaporation [153]. This impairment is characterized by a gradual decrease in the density and diameter of the MGs and an increase in leptomeningeal detachment and obstruction [154]. In both MGD patients and elderly individuals, there is a marked reduction in nonpolar lipids such as cholesteryl esters (ChEs), accompanied by a notable increase in total polar lipids including cholesterol (Ch), (O-acyl)-ω-hydroxy fatty acids (OAHFAs), and free fatty acids (FAs).

Additionally, triglycerides are significantly elevated only in MGD patients. Interestingly, symptom scores indicative of visual disturbances (e.g., blurred vision, glare) demonstrate significant negative correlations with the proportion of nonpolar lipid ChEs, and positive associations with the polar lipids Ch, OAHFA, and FA [155].

Aging-related MGD (ARMGD) can result from numerous MG-related changes that occur with aging, such as reduced proliferation of acinar basal cells, hyperkeratosis, MG atrophy, and ultimately MG detachment. MG also experiences inflammatory cell infiltration with age and changes in innervation patterns, which may also lead to ARMGD [156]. These findings suggest that the aging process of the MGs leads to MGD, which results in tear film instability and may lead to evaporative DED.

Aging has a significant effect on immune system function, particularly in the context of DED, where a drying stimulus can act as a potent effector to induce immune cell infiltration. This immune cell infiltration initiates a vicious cycle of inflammation that exacerbates disease progression [157].

Notably, advancing age is associated with a decline in nearly all innate T-lymphoid receptor (TLR)-induced responses, elevated levels of proinflammatory cytokines, and a diminished capacity of the immune system to regulate immune responses in terms of speed and specificity [25].

Experimental studies have shown a positive correlation between senescence and the infiltration of APCs, especially CD11b+, CD11c+ and MHC II+ cells, on the ocular surface of mice [35, 158, 159]. In both human and mouse conjunctiva, there is an increase in the infiltration of CD4+ T cells, accompanied by the upregulation of Th1- and Th17-related cytokines, such as IFN-γ and IL-17, with age [25].

Immune cells play an active role in safeguarding the integrity of the ocular surface, either through direct mechanisms or via the secretion of immunomodulatory factors. However, dysregulated secretion of these associated factors can lead to inflammation of the ocular surface. In individuals affected by various forms of DED, immune cells infiltrate multiple sites on the ocular surface. This includes immune cell infiltration of the lacrimal gland, elevated levels of inflammatory cytokines in tears, and increased density of immune cells in the cornea and conjunctiva [160].

DED results in corneal epithelial breakdown exposing self-antigens, attracting APCs residing on the ocular surface, delivering ocular surface antigens to naïve T cells and activating them into CD4+ Th1 and Th17 effector cells [161-163]. Notably, aging dendritic cells (DCs) exhibit increased activity in terms of antigen activation, migration, and cytokine production [25].

Increased IFN-γ and IL-17 were found in patients with DED, and in a mouse model, increased IFN-γ promoted cupping apoptosis and the loss of lacrimal gland cells and decreased conjunctival dendritic cell tolerance and ocular surface immunotolerance; however, increased IL-17 led to corneal barrier disruption and lymphangiogenesis [164, 165].

However, while macrophages and other cells can express IFN-γ, the extent to which Th1 cells serve as the primary source of this cytokine in the context of DED remains uncertain. In senescent mice, an observed correlation exists between an increased Th17 population and increased IL-1β expression in initial CD4+ T cells, accompanied by a reduction in IL-2 and IL-2R expression [166].

Th17 cells, especially memory Th17 cells, are significantly increased in the ocular tissues of elderly individuals, which is consistent with the development of chronic ocular surface immune diseases. Inflammatory conditions further contribute to the reduced functionality of Treg cells while promoting the abundance and reactivity of Th17 cells. This imbalance may hinder Treg cell function and expedite the onset of autoimmunity [167, 168]. Therefore, gaining a comprehensive understanding of the Th17/Treg cell equilibrium is crucial for elucidating the underlying mechanisms of DED during the aging process.

Furthermore, elderly patients with DED present elevated levels of proinflammatory cytokines such as IL-1, IL-6, and IL-8. These cytokines trigger the activation of mitogen-activated protein kinases, leading to the secretion of chemokines and matrix metalloproteinases (MMP-3 and MMP-9) in response to hyperosmotic stress, ultimately resulting in apoptosis [160, 169]. As a consequence of this impaired response to infection, immunodeficiency ensues, whereas heightened reactivity to self-antigens leads to chronic inflammation and autoimmunity at the ocular surface.

**Figure 4.**
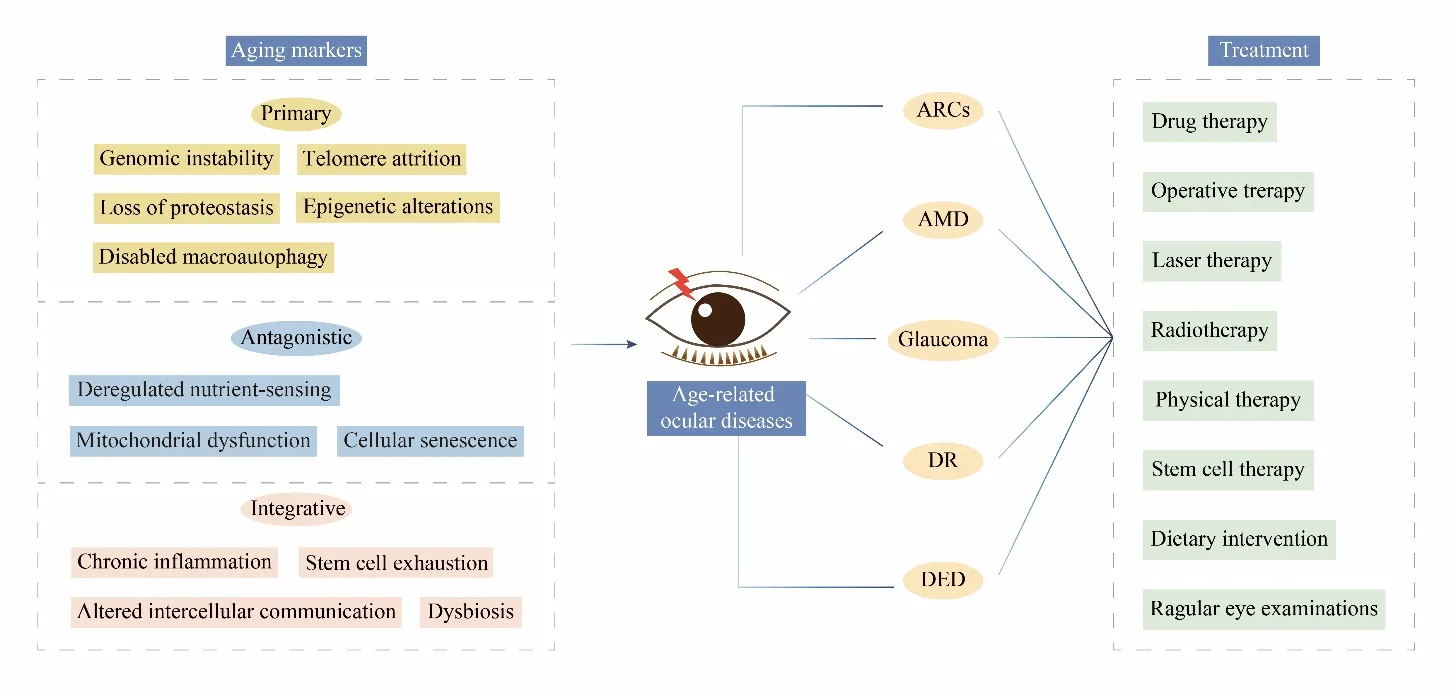
**Aging affects eye diseases and their treatment methods**. Aging markers can be divided into primary markers, antagonistic markers and integrative markers. Aggravating them can promote ageing and affect the occurrence of eye diseases. Treatments for age-related eye diseases include medication, surgery, laser therapy, radiation therapy, stem cell therapy, dietary interventions, and regular eye exams. Abbreviation: ARCs (Age-related cataracts); AMD (age-related macular degeneration); DR (Diabetic retinopathy); DED (dry eye disease).

## 5. Therapeutic Options and Potential Targets for Age-Related Eye Diseases

For age-related eye diseases, conventional treatments typically include pharmacotherapy, surgical intervention, and physical therapy (Fig. 4). Recent advancements in our understanding of the underlying pathophysiology of these conditions have led to the identification of numerous novel therapeutic targets (Table 1). The evolving landscape of treatment options now includes a spectrum of emerging therapies, from antioxidants and anti-inflammatory agents to gene and stem cell therapies, each offering hopes for patients.

**Table 1:** Potential therapeutic targets for age-related eye diseases.

Ocular Diseases	Related targets	Study subject	Mechanism	Ref.
**ARCs**	FOXO3a	HLECs	Overexpression of FOXO3a inhibits cataract production by promoting the TRIM69/p53 pathway.	[174]
	EGCG	C57 mice	EGCG regulates the RASSF2/AKT pathway to delay the development of senile cataract.	[203]
	SIRT6	Male SD rats	Melatonin inhibits ferroptosis by regulating SIRT6/p-Nrf2/GPX4 and SIRT6/NCOA4/FTH1 pathways.	[171]
	FN3K	Equine, Ob/ob mice, patients	FN3K breaks down AGE in the cataract lens, reducing the effect of AGE on the structure and stability of the lens protein.	[172]
	GPX1	C57BL/6J mice	GPX1 maintains the transparency and optical quality of the lens and regulates the homeostasia of the aging lens.	[57]
	circMRE11A	Patients	circMRE11A binds to UBXN1 and activates the ATM/p53/p21 signaling pathway, leading to cell cycle arrest and senescence of LECs.	[204]
	GATA4	HLECs	Up-regulation of GATA4 mediates HLEC dysfunction.	[205]
	Nrf2	C57bl/6 mice, Naked mole-rats	Keap1 activation accelerates Nrf2 proteasomal degradation and eliminates Nrf2 activity, leading to a shortened lifespan.	[206]
	GSTM3	HLECs	Hypermethylation of the GSTM3 promoter and altered histone modifications decrease its expression and promote ARC formation.	[59]
	LMα4	HLE B-3	Increased LMα enhances TGF-β1 levels and activates p38 MAPK signaling during aging, contributing to ARC development.	[58]
**Glaucoma**	Prdx6	Human TM cells	Prdx6 delays aging and restores trabecular reticulum cell health by limiting ROS levels.	[67]
	OPA1	Male Sprague-Dawley rats	Overexpression of OPA1 reduces RGC cytotoxicity and apoptosis while enhancing mitochondrial fusion and autophagy, thereby promoting RGC survival.	[73]
	8-OHG	Adult rhesus monkeys	High levels of 8-OHG lead to neuronal apoptosis and autophagy activation.	[207]
	SPP1	C57BL/6 mice	SPP1 enhances phagocytosis and neurotrophic factor secretion, while inhibiting neurotoxic and pro-inflammatory factor production, thereby mitigating neurodegeneration and preserving visual function.	[208]
	Vitamin B3	DBA/2J (D2) mice	Vitamin B3 has preventive and interventional effects on damage to optic neurons caused by NAD+ decline.	[209]
	Glycosaminoglycans	MPSs patients	Abnormal accumulation of glycosaminoglycans blocks the trabecular meshwork, leading to scleral thickening and altered sieve plate mechanics, which can raise IOP, damage the optic nerve, and cause glaucoma.	[210]
	Sirt6	C57BL/6J mice	Sirt6 reduces attenuated high tension-induced degeneration of RGCs and their axons and prevents RGC senescence.	[74]
	UCP2	C57BL/6J mice	Knockdown of UCP2 promotes mitophagy, thereby reducing oxidative stress and RGC loss.	[82]
**AMD**	APE/REF-1	HRECs	Block abnormal angiogenesis and choroidal neovascularization, thereby improving retinal health.	[183]
	AKT2	Patients, RD8 KO mice	AKT2 upregulation triggers lysosomal dysfunction and mitophagy via the AKT2/SIRT5/TFEB pathway.	[211]
	DAPL1	C57BL/6J mice	DAPL1 deficiency impairs RPE antioxidant defenses and leads to retinal degeneration.	[212]
	amyloid-β	Human RPEs	Accumulation of amyloid-β aggregates leads to increased VEGF expression and ultimately to atrophy and neovascularization in AMD.	[213]
	cGAS-STING	C57BL/6J mice	STING promotes AMD by activating the cGAS-STING pathway, which drives degeneration of RPE and photoreceptor cells.	[214]
	CNTF	hfRPE cells	CNTF binds to CNTFα receptor, activates JAK1 and JAK2, phosphorylates STAT3, which in turn activates gene transcription and enhances the survival of RPEs.	[215]
	RAP1	BN rats	Active RAP1 inhibits choroidal endothelial cell migration and neovascularization by recruiting tight junction proteins and enhancing RPE barrier integrity.	[216]
	COX-2	C57BL/6J mice	COX-2 triggers inflammatory responses, disrupts the blood-ocular barrier, and contributes to choroidal neovascularization in AMD.	[217]
**DR**	Sema4D/PlexinB	male C57BL/6 mice	Sema4D/PlexinB1-mDIA1 signaling affects endothelial cell migration, proliferation, and regulates endothelial cell VE-cadherin phosphorylation leading to vascular leakage.	[218, 219]
	HIFs	ARPE19 cells	High expression of HIF and HIF-regulated vasoactive mediators causes retinal neovascularization and vascular hyperpermeability.	[220]
	Ethanolamine	BV2 cells, Male C57BL/6J mice, GW-DR patients	Ethanolamine supplementation reduces microglia activation and leukocyte adhesion, easing early retinal lesions in diabetic rats.	[221]
	GMFB, SUMO1	ARPE-19 cells	GMFB and SUMO1 expression is significantly elevated in RPE cells in early hyperglycemic state of DM..	[222]
	ADAM10	PDR patients	Impairment of the ADAM10-AXL pathway promotes retinal angiogenesis.	[223]
	TIN2	ARPE-19 cells	The increase of TIN2 can aggravate the aging of RPE cells in vivo and in vitro under the condition of hyperglycemia.	[224]
	circEhmt1	Murine retinal microvascular pericytes and endotheliocytes	circEhmt1-mediated NFIA/NLRP3 pathway activates HIF signaling pathway in pericyte exosomes affecting endothelial angiogenesis.	[225]
	Butylidenephthalide	C57BL/6 mice	BP significantly protected tight junction integrity and RPE cellular physiology through ERK/Nrf-2/HO-1 pathway to prevent DR progression.	[226]
	CGA	HRECs	CGA attenuates diabetic retinopathy by reducing VEGF expression and inhibiting VEGF-mediated retinal neoangiogenesis	[227]
	lipid metabolism	ARPE-19 cells	Oxidative stress mediated by lipid metabolism contributes to high glucose-induced senescence in retinal pigment epithelium.	[228]
	FOXO3a, p53	db/db mice	p53 accelerates endothelial cell senescence in DR by enhancing FoxO3a ubiquitination and UBE2L6 degradation.	[229]
**DED**	IL-20	DED patients	IL-20 promotes macrophage infiltration and activation, leading to severe inflammation and causing DED.	[230]
	FOXO3a	HCE-T cells	FOXO3a/ATG101 stabilizes the ATG13/ULK1 complex and promotes corneal epithelial cell survival.	[231]
	3β-HSD	C57BL/6 mice, non-dry eye/non-MGD volunteers	3β-HSD activity decreases with age and causes MGD.	[232]
	SPARC	Male crossbred dogs	SPARC can promote HCECs repair and tear recovery by enhancing the activity and immunosuppressive effects of ADMSC in vitro.	[233]
	AMPK/SIRT1	C57BL/6J mice, db/db mice	The AMPK-SIRT1 signaling pathway activates autophagy, promotes Nrf2 nuclear translocation, restores antioxidant enzyme activity, and reduces ROS accumulation.	[234]
	TRPM8	C57BL/6 mice	Blockade of TRPM8 alleviates mechanical corneal hyperalgesia and spontaneous ophthalmalgia associated with dry eye.	[235]
	Cathepsin S	C57BL/6 mice	The inhibition of cathepsin S can enhance conjunctival cuprocyte density and corneal mechanosensitivity in aged or Ctss-/- mice.	[236]
	RXRα	Female C57BL/6 mice	RXRα suppresses generation of dry eye disease-inducing IL-17 producing lymphocytes s in the conjunctiva.	[237]

Abbreviation: 3β-HSD, 3β-hydroxyl-steroid dehydrogenase; 8-OHG, 8-hydroxyguanosine; ADMSC, Adipose-derived mesenchymal stem cells; ATG, Autophagy-related; ATM, Ataxia-telangiectasia; BN, Brown norway; BP, Butylidenephthalide; CGA, Chlorogenic acid; CNV, Choroidal neovascularization; CNTF, Ciliary neurotrophic factor; HCECs, Corneal epithelial cell; cGAS-STING, Cyclic GMP-AMP Synthase-Stimulator of Interferon Genes; COX2, cyclooxygenase-2;MDAPL1, Death-associated protein like-1 gene; DM, Diabetes mellitus; GW-DR, DR development in glucose-well-controlled diabetic patients; FOXO3, Forkhead box O3; GMFB Glia maturation factor beta; GSTM3, Glutathione S-Transferase Mu 3; HCE-T, Human corneal epithelial; hfRPE, Human fetal retinal pigment epithelial; HRECs, Human retinal microvascular endothelial cells; ICR, Institute of cancer research; Keap1, Kelch-like ECH-Associated Protein 1; LM, laminin; LECs, lens epithelial cells; MGD, Meibomian gland dysfunction; NAD+, Nicotinamide adenine dinucleotide; Nrf2, Nuclear factor erythroid 2-related factor; Prdx 6, Peroxiredoxin 6; PDR, proliferative DR; Ref-1/APE1, Reduction-oxidation factor 1-apurinic/apyrimidinic endonuclease 1; RGCs, Retinal ganglion cells; RPE, retinal pigment epithelial; SPARC, Secreted Protein Acidic and Rich in Cysteine; Sirts 6, Sirtuins 6; SUMO, small ubiquitin-related modifier; sSema4D, soluble semaphorin 4D; SPP1, secreted phosphoprotein 1; TM, Trabecular meshwork; TFEB/E3, Transcription factor EB/E3; TGF-β1, Transforming growth factor-beta1; TRPM8, Transient receptor potential melastatin 8; TIN2, TRF1-interacting protein 2; UBXN1, UBX domain-containing protein 1; ULK1, unc-51-like kinase 1; UCP2, Uncoupling protein 2.

Currently, surgical removal of the lens and replacement with an intraocular lens (IOL) is the standard treatment for ARCs, yielding significant therapeutic effects. Nevertheless, research into small-molecule strategies for the management of ARCs is lacking.

Epigallocatechin gallate (EGCG), one of the most potent antioxidants present in green tea, has the ability to delay the onset of ARCs by modulating the RASSF2/AKT pathway. Specifically, RASSF2 mediates the inhibitory effect of EGCG on lens epithelial cell apoptosis and the development of ARCs by manipulating the phosphorylation of AKT (Ser473) [170].

Melatonin can inhibit ferroptosis and delay the onset of ARCs by regulating the SIRT6/p-Nrf2/GPX4 and SIRT6/NCOA4/FTH1 pathways [171]. Additionally, fructosamine-3-kinase (FN3K) reduces the impacts of AGEs on lens protein structure and stability by breaking them down in cataractous lenses. Injection or ocular application of FN3K can enhance these effects. However, further studies are needed to establish the efficacy and safety of FN3K for cataract treatment [172].

FOXO3a protein, as an antioxidant transcription factor, plays an important role in regulating the aging process. Overexpression of FOXO3a significantly enhances the activity of the TRIM69 promoter, which induces the ubiquitination of p53, inhibits UVB-induced apoptosis of HLEC and ROS generation, and consequently inhibits cataract formation. Resveratrol can prevent oxidative stress-induced cell death in lens epithelial cell culture by increasing the expression of FOXO1a, FOXO3a and FOXO4 [173, 174].

Treatment options for glaucoma can be categorized into three main groups: laser therapy, surgical therapy, and pharmacologic therapy [175]. Currently, therapeutic strategies focus primarily on reducing IOP through various approaches such as decreasing atrial aqueous production, enhancing scleral outflow, or increasing trabecular meshwork outflow [176]. Pharmacologic therapy plays a crucial role in the treatment of glaucoma, and numerous trials are underway to evaluate both traditional and novel pharmacologic treatments.

Prostaglandin analogues (PGAs), for example, effectively lower IOP by targeting prostaglandin F receptors (PTGFRs) and prostaglandin E receptors (PTGER 1-4), thereby facilitating atrial aqueous outflow, particularly via the uveal pathway [177, 178]. Furthermore, mouse experiments have shown promising results with a small molecule inhibitor of VE-PTP. This inhibitor activates Tie2 within Schlemm's canal, resulting in an increased filtered area and improved outflow ability, consequently leading to reduced IOP [179].

Another potential avenue for glaucoma treatment involves addressing the age-dependent decline in nicotinamide adenine dinucleotide (NAD) levels in the retina. NAD is a critical molecule for maintaining mitochondrial health, and its decline renders RGCs more susceptible to damage during periods of elevated IOP. Therefore, supplementation with NAD has neuroprotective effects and has therapeutic potential for treating glaucoma [180, 181].

AMD is a complex disease, and researchers have conducted extensive research to elucidate its pathogenesis to identify effective therapeutic approaches. Ongoing clinical trials explore a diverse range of therapeutic categories, including complement pathway inhibitors, visual cycle modulators, toxic byproduct reduction, antioxidant therapy, neuroprotective agents, laser therapy, surgical therapy, gene therapy, stem cell therapy, and other innovative therapies [182].

Oxidative stress is a key contributor to AMD pathogenesis, and reducing oxidative stress has been shown to effectively mitigate disease progression. Apurinic/apyrimidinic endonuclease/redox effector factor-1 (APE/REF-1) is a multifunctional protein that exhibits redox activity and DNA repair function through its involvement in retinal angiogenesis, endothelial cell proliferation, migration, and tube formation. APE/REF-1 inhibitors block abnormal angiogenesis and choroidal neovascularization, thereby improving retinal health [183, 184].

Several clinical trials have demonstrated that supplementation with zeaxanthin and lutein improves visual function, attenuates VEGF-induced neovascularization in human retinal microvascular endothelial cells, and reduces the risk of progression to advanced AMD [185, 186].

However, the efficacy of zeaxanthin and lutein in preventing ocular disease or improving visual function remains uncertain, as some studies have not found a significant correlation [187]. This variability underscores the possibility that the effectiveness of these carotenoids may be influenced by factors such as specific populations, dosage levels, and the stages of disease [188]. Therefore, further comprehensive research is needed to clarify their potential benefits in retinal diseases.

Arbutin, cotton-phenol acetic acid, and vitamin C find applications in the treatment of AMD by regulating the expression and nuclear translocation of FOXO3a, enhancing the transcriptional activity of FOXO3a, and resisting oxidative stress in RPE [189]. Arbutin has shown potential therapeutic effects on AMD only in mouse models, and no relevant clinical trials are available. Whereas the inhibitory effect of vitamin C on AMD has been validated in the US population, its general applicability in other populations still requires further study.

Mitochondria play a crucial role in AMD, and the mitochondrion-targeted antioxidant SS-31 scavenges ROS produced by the inner mitochondrial membrane, thereby reducing mitochondrial ROS and exerting a significant cytoprotective effect on the ocular cell line against hydrogen peroxide-induced chronic oxidative stress [111, 184]. Moreover, pluripotent stem cell transplantation of RPE cells has emerged as a promising strategy for treating AMD. Loriana Vitillo et al. demonstrated improved results via this approach [190].

DR is strongly associated with diabetes, with a global prevalence exceeding 100 million individuals [191]. Common treatment options currently available include laser photocoagulation, intravitreal injections of VEGF antagonists (anti-VEGF), steroids, and vitreoretinal surgery [129]. Currently, anti-VEGF drugs are considered the primary treatment for DR. However, frequent injections impose a greater financial burden on patients, making this treatment less than ideal [192]. In contrast, targeted retinal photocoagulation (TRP) is an emerging laser therapy that specifically targets unperfused retinal capillaries and intermediate ischemic regions, thereby reducing damage to healthy tissue and reducing complications or adverse effects of panretinal photocoagulation (PRP). Furthermore, the intravitreal injection regimen of TRP in combination with anti-VEGF drugs also offers a new treatment option for DR [193].

Recent studies have identified microparticles as potential therapeutic targets in the early stages of DR and vascular aging. These microparticles respond to various stressors and stimuli during shedding and are produced during apoptosis.

They can transport a range of macromolecules, including factor XII, tissue factor, or mitogen-activated protein kinase, which possess neoangiogenic properties. Additionally, microparticles carry miRNAs involved in the regulation of vascular senescence or remodeling [194]. A detailed understanding of the mechanisms underlying microparticle release may greatly aid in the development of effective treatments for DR.

Curcumin is a natural compound that has been demonstrated in animal studies to process anti-apoptotic, anti-inflammatory, antioxidant, anti-cancer and vascular function effects. In recent years, studies have also confirmed that curcumin can prevent multiple diabetes complications by improving glucose and lipid metabolism, increasing insulin sensitivity, reducing insulin resistance, and ameliorating oxidative stress and inflammatory pathway status in both diabetes and DR. Nevertheless, further experiments need to be designed to evaluate whether it can become a novel drug for the treatment of DR in clinical practice [195, 196].

Excess O-GlcNAc modification has been implicated in insulin resistance, vascular cell death, disruption of endothelial cell integrity, neovascularization, and neurodegeneration, all of which play significant roles in various stages of DR. Therefore, this modification holds promise for the early diagnosis of DR and the development of novel precision therapies [131].

Currently, DED cannot be completely cured, and the most commonly used approach is the application of artificial tears to moisturize the ocular surface. These drops serve to hydrate DEDs, lubricate the ocular surface, and mitigate inflammation by diluting inflammatory factors, thereby lowering osmolarity and reducing sensitivity to inflammation [197]. Depending on the severity of DED, various medications can be used for intervention, including anti-inflammatory agents and topical mucus secretion agents [198].

In cases of advanced and severe DED, autologous serum from patients can be utilized as a therapeutic option. Autologous serum contains growth factors, immunoglobulins, vitamin A, and other beneficial substances, and has demonstrated efficacy in managing advanced DED. However, it is worth noting that the preparation of autologous serum is a complex process and is prone to contamination [198].

Anti-inflammatory agents, including cyclosporine, are commonly employed in the treatment of DED. Cyclosporine is a fungus-derived peptide that has demonstrated anti-inflammatory and immunosuppressive properties by inhibiting T-cell activation and the production of inflammatory cytokines [199]. For the management of severe DED, glucocorticoids such as hydrocortisone may be used for brief periods to control inflammation, with caution exercised to avoid prolonged use due to potential corticosteroid-related side effects [165].

Some animal experiments have shown that the gut microbiota can modulate the severity of ocular surface inflammation and DED; thus, the gut microbiota could be a potential therapeutic target for DED [200].

Currently, therapeutic strategies focus more on active tear production rather than tear replacement. For example, researchers are currently investigating the use of neurostimulant drugs to chemically stimulate the lacrimal gland and promote the production of aqueous tears. Additionally, certain drugs act as mucin secretagogues, stimulating increased mucin secretion from conjunctival goblet cells and/or increasing the number of these cells. One example is the P2Y2 purinergic receptor agonist diquafosol [201].

Monitoring the effects of aging on eye diseases is critical in clinical management. For the elderly population, a fundus examination at least once a year is recommended for early identification of early signs of AMD and DR. In individuals diagnosed with glaucoma, intraocular pressure should be monitored regularly and combined with optic nerve head analysis to monitor the progression of the disease. Management of dry eye can be done by evaluating tear secretion and tear film stability, and regular evaluation and use of artificial tears or other treatments can reduce symptoms. In addition, older adults should be encouraged to adopt healthy lifestyles, such as increasing antioxidant intake and managing chronic diseases, to reduce the risk of eye diseases.

## 6. Conclusion

In summary, aging represents a significant risk factor for the development of ocular diseases. The functional and structural degeneration of ocular tissues, alongside inflammatory responses, apoptosis, and DNA damage are pivotal contributors to the onset and progression of these conditions. Aging precipitates a decrease in the body's antioxidant capabilities, resulting in cellular oxidative stress. This stress leads to age-related ocular manifestations, including cataracts, which are characterized by lens clouding, and AMD, which is characterized by diminished retinal pigment epithelial cell activity and increased mortality.

Furthermore, prolonged hyperglycemia catalyses ROS accumulation and diminishes antioxidant function within the aging retina, thereby facilitating the development of DR. Aging also impacts mitochondrial function; mitochondrial dysfunction, in turn, promotes ROS accumulation, triggering trabecular meshwork cell apoptosis. These events increase resistance to the outflow of aqueous humor, culminating in a pathological increase in IOP and the induction of glaucoma. Concurrently, the combined effects of aging and inflammation destabilize the tear film, rendering it hyperosmotic and initiating a vicious cycle of inflammation in dry eye syndrome, which exacerbates disease progression.

Future research on eye diseases and aging should concentrate on several key domains. Firstly, the molecular and cellular mechanisms related to aging are probed into profoundly to disclose their impacts on eye tissue and function, encompassing the deterioration of retinal and lens cell function resulting from cellular aging, and the augmentation of oxidative stress and inflammatory response leading to cell damage and tissue degradation. These mechanisms can assist in uncovering biomarkers for novel therapeutic targets, thereby attaining early detection and diagnosis of diseases.

As we acquire a deeper comprehension of cellular aging mechanisms, researchers will strive to develop a broader spectrum of drugs and treatments, such as the development of novel antioxidants, anti-inflammatory drugs and cell protectants. And the utilization of gene editing technology and stem cell therapy to repair or substitute damaged eye cells.

Early detection and intervention are critical to preventing the deterioration of eye diseases. Researchers have discovered that liquid biopsy proteomics can be integrated with artificial intelligence to identify cellular drivers of eye aging and disease within the body [202]. In addition, bioinformatics techniques, such as genomics and transcriptomics, can be employed to identify key genes and regulatory networks associated with age-related eye diseases. By comparing gene expression patterns in young and old individuals, potential biomarkers and therapeutic targets can be identified to facilitate prevention and treatment. Future research should leverage the strengths of interdisciplinary collaboration, incorporating bioinformatics, systems biology, gerontology and other majors, jointly addressing the complex issues in the detection and treatment of eye diseases.

The influence of aging on eye health varies significantly worldwide, influenced by demographics, healthcare systems, and access to care. In developed countries, for instance, advanced medical technologies and screening procedures have made the early diagnosis and management of diseases such as AMD and glaucoma more effective. Nevertheless, developing countries may confront a higher burden of disease and less efficient management due to uneven healthcare resources. In some regions, limited health education and resources can result in the early signs of eye diseases being overlooked, escalating the risk of visual impairment. Globally, differences in healthcare policies and interventions have a direct effect on the prevalence and treatment outcomes of eye diseases in older adults. Therefore, developing regionally targeted screening and interventions, as well as enhancing access to medical resources globally, are important steps to improve eye health in older adults.

Inevitably, the current manuscript possesses certain limitations. Firstly, disparities exist in the study design and methodology of the incorporated studies, and variations in sample sizes, populations, as well as diagnostic criteria might result in incongruities in the reported mechanisms and treatment outcomes. Additionally, this review mainly concentrates on published literature and might exclude relevant unpublished data or ongoing studies that could offer additional insights. The conclusions could also be affected to publication bias, with a higher probability of statistically significant results being published, which might exaggerate the role of aging in ocular disease. Eventually, although we endeavored to cover a wide range of ocular diseases, we failed to include all ocular diseases that might be associated with aging, such as fugax and presbyopia. Future research ought to bridge these gaps by encompassing a broader spectrum of diseases, adopting more rigorous study designs, and integrating published and unpublished findings to acquire a more comprehensive comprehension of the interactions between aging and ocular disease.

While the process of aging remains beyond our control, advancing our understanding of its molecular underpinnings in relation to ocular diseases may hold promise for the development of novel therapeutic interventions.
